# Candidate DNA methylation markers for gestational diabetes: findings from technical and biological validation in a South African population

**DOI:** 10.1007/s00438-026-02528-7

**Published:** 2026-10-26

**Authors:** Stephanie Dias, Matladi Masete, Sumaiya Adam, Hygon Mutavhatsindi, Carmen Pheiffer

**Affiliations:** 1 Biomedical Research and Innovation Platform (BRIP), South African Medical Research Council, Tygerberg, Cape Town, 7505 South Africa; 2 Department of Obstetrics and Gynaecology, School of Medicine, Faculty of Health Sciences, University of Pretoria, Pretoria, 0001 South Africa; 3 Diabetes Research Centre, Faculty of Health Sciences, University of Pretoria, Pretoria, 0001 South Africa; 4 Department of Medicine, Faculty of Health Sciences, University of Cape Town, Cape Town, 7925 South Africa; 5 Non-Communicable Diseases Research Unit (NCDRU), South African Medical Research Council, Cape Town, 7505 South Africa

**Keywords:** DNA methylation, Biomarkers, Gestational diabetes mellitus, Pregnancy, Epigenetics

## Abstract

Gestational diabetes mellitus (GDM) increases risk of adverse maternal and fetal outcomes. Using Genome-wide Beadchip arrays, we previously reported differential methylation of 1046 CpG sites in women with GDM. This study aimed to validate three candidate CpG sites in an independent cohort of South African women with and without GDM. DNA methylation was assessed in maternal peripheral blood collected at < 28 weeks of gestation, at three top-most significant CpG sites within Calmodulin Binding Transcription Activator 1 (*CAMTA1)*, Kelch domain-containing protein 3/ Male-Enhanced Antigen 1 (*KLHDC3/MEA1)*, and Collectin Subfamily member 10 (*COLECT10)*, using bisulfite pyrosequencing, in a technical validation group (12 GDM and 12 non-GDM), and an independent biological validation group (46 GDM and 135 non-GDM). Associations between differentially methylated CpGs and maternal clinical characteristics were assessed. Technical validation confirmed decreased methylation at *cg23643951* (*CAMTA1*; -1.53-fold; *p* = 0.04) and *cg21910650 (KLHDC3/MEA1;* -1.57-fold; *p* = 0.004), and increased methylation at *cg16306629* (*COLECT10;* 4.5-fold; *p* = 0.003) in women with GDM, consistent with the original array findings. No significant differences were observed in the biological validation group. Within the technical validation group, *cg21910650* methylation was inversely correlated with fasting glucose (rho=-0.625; *p* = 0.015) and 1 h OGTT (rho=-0.555; *p* = 0.034), while *cg16306629* was positively correlated with fasting glucose (rho = 0.709; *p* = 0.006). Technical validation confirmed differential methylation at three CpG sites, though these findings were not reproduced in the larger biological validation group, highlighting the need for validation in independent cohorts. Associations between CpG methylation with glucose concentrations suggest a potential association with glucose regulation during pregnancy, providing a foundation for future validation of these CpG sites as biomarkers for GDM.

**Supplementary Information:**

The online version contains supplementary material available at https://doi.org/10.1007/s00438-026-02528-7.

## Highlights

- Technical validation confirmed decreased methylation at *cg23643951* within *CAMTA1* and *cg21910650* within *KLHDC3/MEA1*, and increased methylation at *cg16306629* nearest to *COLECT10* in women with GDM in this population, consistent with original array findings
- Two of these CpG sites were associated with glucose concentrations, suggesting a possible role in glucose tolerance during pregnancy.
- These findings were not reproduced in the larger independent biological validation cohort, highlighting challenges in translating methylation findings into clinically useful biomarkers.
- This study provides a foundation for future research to validate the clinical utility of these three CpG sites as biomarkers for GDM.

**Supplementary Information:**

The online version contains supplementary material available at https://doi.org/10.1007/s00438-026-02528-7.

## Introduction

Gestational diabetes mellitus (GDM), defined as glucose intolerance first diagnosed during pregnancy, affects approximately 16% of pregnancies worldwide and is increasing globally, posing a significant health risk for both mother and child (IDF 2025). The prevalence of GDM varies significantly across countries, with recent estimates reporting a 10.4% prevalence in Africa (IDF 2025). The risk of GDM is further exacerbated by factors such as obesity, advanced maternal age, excessive weight gain during pregnancy, previous GDM, and a family history of diabetes (Choudhury and Devi Rajeswari 2021). Without timely diagnosis and appropriate glucose management, GDM is associated with both short- and long-term adverse outcomes for mother and child. Short-term complications include preeclampsia, cesarean delivery, macrosomia, hypoglycemia, and birth trauma (Negrato et al. 2012; Muche et al. 2020). In the long term, women with GDM have a 8 to 10-fold increase risk of developing type 2 diabetes (T2DM) and a 2-fold higher risk of cardiovascular disease (CVD) compared to women without prior GDM (Adam et al. 2023), while infants have an increased risk of future obesity and metabolic disorders (Burlina et al. 2019). The identification of women with GDM who are at risk of developing adverse outcomes allows the introduction of timely management and intervention strategies to prevent or improve maternal and infant health outcomes. Thus, research efforts aimed at identifying reliable, accessible biomarkers for the early detection and management of GDM are increasingly being explored.

Epigenetics mechanisms, particularly DNA methylation have been implicated in the pathophysiology of metabolic disease, including GDM (Dias et al. 2022). DNA methylation is a reversible process involving the addition of a methyl group to the fifth carbon (C5) of cytosine dinucleotides to create 5-methylcytosine (5mC) (Jones 2012), and is known to regulate gene expression. These modifications have been shown to be altered in genes central to glucose homeostasis, insulin signaling, and β-cell function, and when dysregulated, may contribute to metabolic disease (Fradin et al. 2012; Gilbert and Liu 2012). Accordingly, high-throughput genome-wide studies using Illumina BeadChip technologies in maternal peripheral blood, placenta and cord blood of women with and without GDM have identified several differentially methylated regions and loci associated with genes and pathways relevant to GDM pathophysiology (Enquobahrie et al. 2015; Finer et al. 2015; Haertle et al. 2017; Kang et al. 2017). Importantly, physiological and DNA methylation changes that occur during pregnancy have been shown to be reflected in whole blood (Fradin et al. 2023), increasing interest in screening maternal blood for biomarkers of GDM. Profiling DNA methylation in peripheral blood may therefore offer a feasible, minimally invasive approach to monitor high-risk individuals and facilitate early intervention, supporting its potential as a biomarker for GDM. However, translation of these findings into clinically useful biomarkers remains limited. Targeted methylation assays such as bisulfite pyrosequencing, widely regarded as the gold standard for methylation quantification, are increasingly being explored as a method used to validate genome-wide methylation findings. Accordingly, several studies across diverse populations such as American, Chinese, European and South Asian have validated their findings in peripheral blood of women with GDM (Binder et al. 2015; Rong et al. 2015; Deng et al. 2018; Wu et al. 2018; Yan et al. 2021; Linares-Pineda et al. 2023), yet African populations remain significantly under-represented. In our previous genome-wide methylation study conducted in the peripheral blood of South African women with (*n* = 12) and without GDM (*n* = 12), we identified 1046 differentially methylated CpG sites mapped to 939 genes enriched in GDM-related metabolic pathways. Of these, 148 were shown to be hypermethylated, while 898 were hypomethylated in women with GDM compared to women without GDM (Dias et al. 2019). The aim of this study was to validate three of the most significant candidate CpG sites, *cg23643951* within Calmodulin Binding Transcription Activator 1 (*CAMTA1)*, *cg21910650* overlapping Kelch domain-containing protein 3 and Male-Enhanced Antigen 1 (*KLHDC3/MEA1)*, and *cg16306629* nearest to Collectin Subfamily member 10 (*COLECT10)*, using bisulfite pyrosequencing. Validation was performed in two stages. First, technical validation was conducted in the same 24 women with and without GDM from the original genome-wide study, to confirm concordance between array-based and pyrosequencing-based differential methylation measurements at the three candidate CpG sites. Second, biological validation was conducted in an independent sample set comprising 46 women with GDM and 135 women without GDM who had not been included in the genome-wide study, to determine reproducibility of differentially methylated CpG sites. In addition, associations between differentially methylated CpGs and maternal clinical characteristics were assessed.

##  Materials and methods

### Participants

The study was conducted according to the Declaration of Helsinki and all women gave written informed voluntary consent after the procedures had been fully explained. The study included two sample sets for validation: A technical validation group comprising the same 24 women (with GDM (*n* = 12) and without GDM (*n* = 12) previously selected for the BeadChip array analysis (Dias et al. 2019), matched according to age, body mass index (BMI) and gestational age as far as possible, and a larger independent biological validation group comprising women with GDM (*n* = 46) and without GDM (*n* = 135), who had not been included in the original genome-wide study.

Participants were selected from a prospective cohort study conducted at a primary healthcare clinic in Johannesburg, Gauteng, South Africa. Participants were recruited at less than 28 weeks of gestation and were included in the study if they had singleton pregnancy, were self-reported black ethnicity, were between the ages of 18 and 40 years and were human immunodeficiency virus (HIV) negative. At the first visit, random glucose and glycated hemoglobin (HbA1c) levels were measured using a glucometer (Roche Diagnostics, Mannheim, Germany) and the point-of-care Afinion system (Alere Technologies, Oslo, Norway), respectively. Women with random glucose and HbA1c concentrations more than 11.1 mmol/L and 6.5% were excluded from the study. Women who met the inclusion criteria were requested to return in a fasted state for blood collection prior to GDM diagnosis. Thereafter, GDM was diagnosed between 24 and 28 weeks of pregnancy using the 75 g 2 h OGTT according to the International Association of the Diabetes and Pregnancy Study Groups criteria (IADPSG) (International Association of Diabetes and Pregnancy Study Groups Consensus Panel 2010). Demographic information was obtained from a standardised questionnaire, and anthropometric measurements were assessed according to standard hospital procedures (Adam et al. 2018). Fasting plasma glucose, and insulin concentrations were measured in an accredited laboratory (Vermaak and partners (Pretoria)/PathCare (Cape Town) Laboratories, South Africa).

### DNA extraction and quantification

DNA were extracted from 2 ml of peripheral blood collected at less than 28 weeks of gestation, using the QIAamp DNA Blood Midi Kit (Qiagen, Hilden, Germany), according to manufacturer’s instructions. The quantity and quality of DNA were assessed using the NanoDrop ^®^ ND-1000 instrument (Nanodrop Technologies, Wilmington, USA), and the Qubit Fluorometer (ThermoFisher Scientific, Waltham, USA) with the Quanti-iT dsDNA Broad Range assay kit (ThermoFisher, Massachusetts, USA), according to the manufacturers’ instructions.

### Primer design

**Fig. 1:**
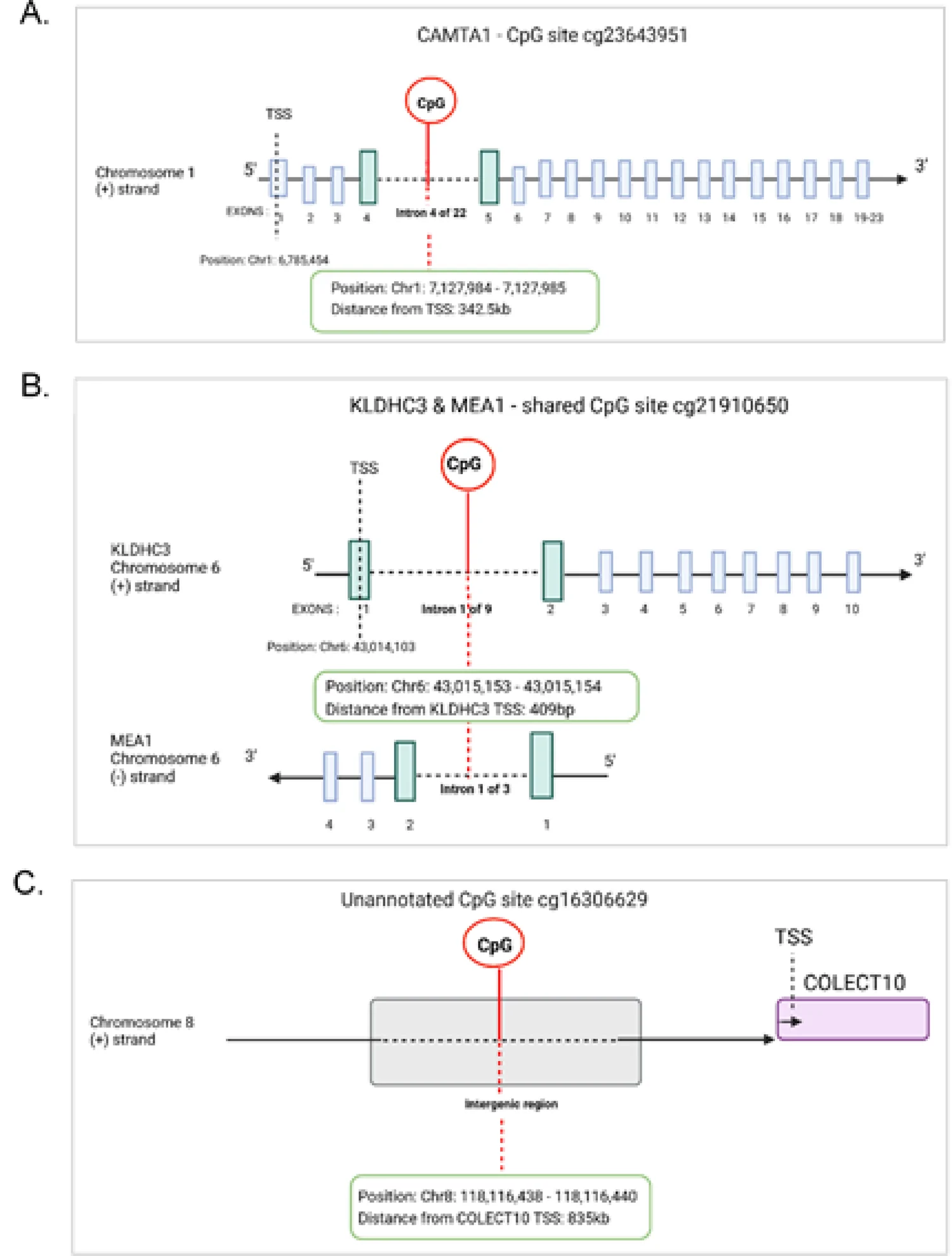
An overview of the genomic location of the selected CpG sites, *cg23643951*, *cg21910650* and cg16306629. **A** *cg23643951* is located on Chr 1:7,127,984-7,127,984 in intron 4 of *CAMTA1*, 342.5 kb downstream from the TSS, **B** *cg21910650* is located on Chr 6: 43,015,153 − 43,015,154 overlapping *KLHDC3/MEA1* in intron 1 on the + and – strand, respectively, 409 bp downstream from the TSS and **C** *cg16306629* is an unannotated CpG site, located on Chr 8: 118,116,438 − 118,116,439 in an intergenic region, 385 kb upstream from *COLECT10* TSS (nearest regulatory region). Abbreviations: TSS, Transcription start site; Chr. Chromosome (Created with BioRender.com

Three significant CpG sites, *cg23643951* within *CAMTA1*,* cg21910650* overlapping Kelch domain-containing protein 3 and Male-Enhanced Antigen 1 (*KLHDC3/MEA1)*, and one unannotated CpG, *cg16306629*, with *COLECT10* as the nearest gene regulatory region, were selected from our previous genome-wide methylation study (Dias et al. 2019) for validation analysis. An overview of the genomic locations of the selected CpG sites relative to the hg38 human genome reference database, including their positions within genes and intergenic regions, is shown in Fig. 1. Primers were designed using the PyroMark Assay Design Software (version 2.0.2.5, Qiagen) and the UCSC Genome Browser (GRCh38/hg38) (https://genome.ucsc.edu/cgi-bin/hgGateway). Details for the primer and target sequence are shown in Table S1.

###  DNA methylation

Briefly, 500 ng of DNA was bisulfite converted using the EpiTech Fast DNA Bisulfite Kit (Qiagen, Hilden, Germany), according to the manufacturer’s instructions. Quantitative real-time PCR (qRT-PCR) was performed on 20 ng of bisulfite converted DNA using the Pyromark PCR kit and pyrosequencing was conducted using the PyroMark Gold Q96 kit and the PyroMark Q96 pyrosequencer (Qiagen). Pyrosequencing generates a quantitative measure of percent methylation at each individual CpG site, calculated from the ratio of cytosine (C) to the sum of cytosine and thymine (C + T) peak heights at that site, using PyroMark Q96 software (Qiagen). Bisulfite conversion efficiency and assay performance were evaluated using methylation standards (0%, 10%, 25%, 50%, 75% and 100% methylation) for assessing *CAMTA1*, *KLHDC3/MEA1* and *COLECT10* (S1 Fig). Samples that did not pass pyrosequencing QC were omitted from analysis.

### Statistical analysis

Participant characteristics were tested for normality using the Shapiro-Wilk test in STATA 14 (StataCorp, College Station, USA) and GraphPad Prism version 5.0 (GraphPad Software, Inc., La Jolla, CA, USA). Normally distributed data are presented as the mean ± standard deviation (SD) or as the median and interquartile range (IQR) for data that were not normally distributed. An unpaired t tests (normal distributed) or the Mann-Whitney tests (non-normal distribution) were used to compare clinical and CpG specific methylation differences between GDM and non-GDM women. The correlation between differentially methylated CpG sites and participant clinical characteristics were assessed using Spearman’s correlation. A *p* < 0.05 was considered statistically significant.

## Results

### Participant characteristics

Participant characteristics are presented in Table 1. Women in the technical validation group were matched for age, BMI and gestational age, and as expected, no significant differences were observed between women with and without GDM. In contrast, women in the biological validation group were not matched, and while age and BMI did not differ between women with and without GDM, gestational age was lower among women with GDM compared to women without GDM (*p* = 0.035). In the technical validation group higher fasting (*p* < 0.001) and 1 h OGTT glucose concentrations (*p* = 0.006) were observed in women with GDM compared to women without GDM. In the biological validation group, women with GDM had higher random glucose (*p* < 0.001), 1 h OGTT (*p* < 0.001), 2 h OGTT (*p* < 0.001), and HbA1c (*p* = 0.015) levels, and lower adiponectin concentrations (*p* = 0.043) compared to women without GDM. Fasting insulin and CRP did not differ significantly between groups in either validation set.

**Table 1:** Participant characteristics

Participant Characteristics	Technical validation			Biological validation		
	Non-GDM	GDM	*p*-value	Non-GDM	GDM	*p*-value
Participants: n	12	12		135	46	
Age (years)	27.3 ± 1.1	27.3 ± 1.1	1.000	26.0 (21.0–31.0)	27.0 (24.8–33.0)	0.097
BMI (kg/m^2^)	27.1 ± 4.1	27.6 ± 3.9	0.774	26.3 (23.5–29.9)	27.7 (22.9–32.8)	0.276
Gestational Age (weeks)	27.5 (27.0–28.0)	27.0 (26.0–28.0)	0.511	25.0 (22.0–27.0)	24.5 (21.0–26.0)	**0.035**
HbA1c (%)	5.2 ± 0.4	5.1 ± 0.4	0.853	5.1 ± 0.4	5.3 ± 0.3	**0.015**
Random glucose (mmol/L)	4.3 ± 0.6	4.6 ± 0.5	0.182	4.3 (4.0-4.7)	4.9 (4.3–5.1)	**< 0.001**
Fasting plasma glucose (mmol/L)	4.3 ± 0.3	5.5 ± 0.4	**< 0.001**	4.3 (4.0-4.6)	5.5 (5.2–5.9)	**< 0.001**
1 h OGTT (mmol/L)	5.2 (4.5–5.8)	6.2 (5.7–7.9)	**0.006**	5.6 ± 1.3	6.7 ± 1.7	**< 0.001**
2 h OGTT (mmol/L)	5.3 ± 0.8	5.8 ± 1.0	0.152	5.3 (4.7–5.9)	5.9 (5.1–7.1)	**< 0.001**
Fasting insulin (mIU/mL)	8.0 (6.7–10.1)	10.2 (6.3–12.7)	0.678	5.9 (4.2–8.4)	6.5 (5.0-8.7)	0.174
CRP (mg/L)	7.1 ± 3.0	7.7 ± 3.6	0.750	7.1 (3.8–8.6)	7.5 (3.7–11.2)	0.470
Adiponectin (µg/ml)	14.3 ± 9.6	8.8 ± 4.6	0.129	10.0 (7.2–14.4)	7.9 (5.1–10.7)	**0.043**

Data are expressed as the mean ± standard deviation (SD) for normally distributed data and median interquartile range (IQR) for data that are not normally distributed data. Differences between groups were determined using the unpaired student *t* test or the Mann-Whitney test. Abbreviations: BMI: body mass index, OGTT: oral glucose tolerance test, HbA1c: glycated hemoglobin, CRP: C-reactive protein. *p* < 0.05 was considered statistically significant.

### 3.2 Validation of differential methylation at candidate CpG sites

**Fig. 2:**
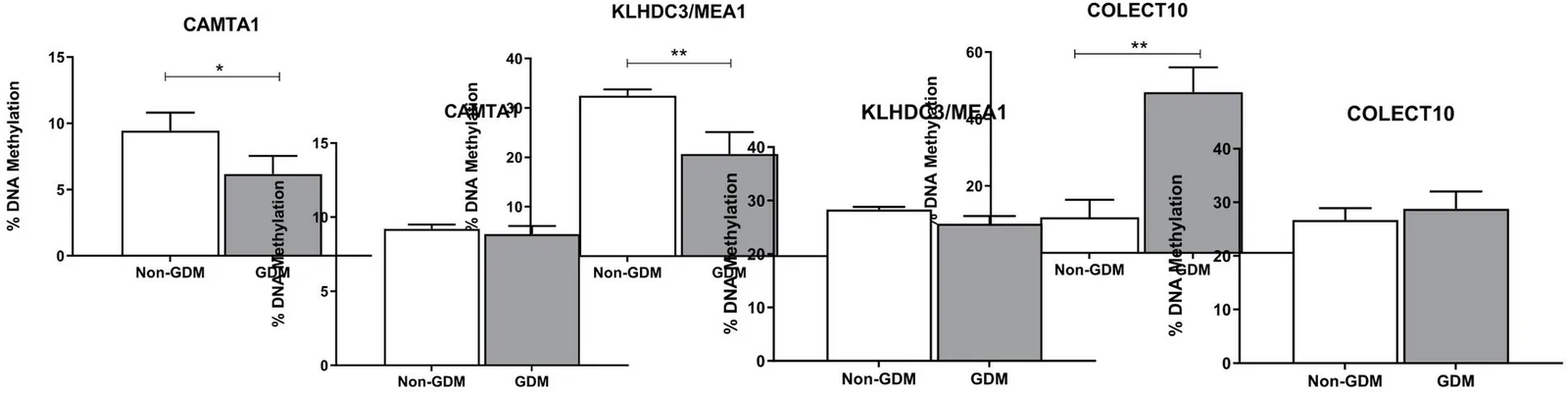
Methylation levels at candidate CpG sites in women with and without GDM. Pyrosequencing validation of three CpG sites associated with *CAMTA1*,* KLHDC3/MEA1*, and *COLECT10* in maternal whole blood for the: (A–C) Technical validation group and (D–F) Biological validation group. *COLECT10* is the nearest gene regulatory region to the CpG site, cg16306629, that lies within an intergenic region. *p* < 0.05^*^, *p* < 0.01^**^ indicates statistical significance

Three CpG sites identified from the BeadChip array data, associated with *CAMTA1*, *KLHDC3/ MEA1*, and an intergenic region near *COLECT10* regulatory region, were successfully optimised for pyrosequencing analysis (Figure S1). Technical validation confirmed decreased methylation levels at cg23643951 in *CAMTA1* (-1.53-fold; *p* = 0.040) and cg21910650 in *KLHDC3/MEA1* (-1.57-fold; *p* = 0.004), and increased methylation levels at *cg16306629* near *COLECT10* (4.5-fold; *p* = 0.003), in women with GDM compared to women without GDM. In contrast, no significant differences were observed at any of the three CpG sites in the independent biological validation group (Fig. 2).

###  Correlation between differentially methylated CpG sites and participant characteristics

Significant differential methylation findings identified in the technical validation group prompted further investigation into their association with clinical parameters (Table 2). Methylation levels at *cg21910650* within *KLHDC3/MEA1* were inversely associated with fasting plasma glucose (rho: -0.625, *p* = 0.015) and 1 h OGTT levels (rho: -0.555, *p* = 0.034), while cg16306629 closest to *COLEC10* was positively associated with fasting plasma glucose levels (rho: 0.709, *p* = 0.006).

**Table 2:** Spearman’s correlation between differentially methylated CpG sites in the technical validation group and participant characteristics

Variable	cg23643951 (CAMTA1)		cg21910650 (KLHDC3/MEA1)		cg16306629 (COLEC10)	
	rho	*p*-value	rho	*p*-value	rho	*p*-value
HbA1c (%)	–0.065	0.814	0.169	0.545	–0.221	0.444
Random glucose (mmol/L)	–0.354	0.194	–0.250	0.366	0.468	0.092
Fasting plasma glucose (mmol/L)	-0.422	0.118	–0.625	**0.015**	0.709	**0.006**
1 h OGTT (mmol/L)	0.081	0.773	–0.555	**0.034**	0.435	0.120
2 h OGTT (mmol/L)	0.356	0.210	0.191	0.510	–0.088	0.773
Fasting insulin (mIU/mL)	–0.012	0.162	–0.190	0.603	0.360	0.340
CRP (mg/L)	–0.480	0.980	–0.092	0.809	–0.075	0.854
Adiponectin (µg/ml)	0.311	0.411	0.288	0.458	–0.571	0.151

## Discussion

DNA methylation has been implicated in the pathogenesis of various metabolic diseases and has attracted considerable interest as potential biomarkers for GDM (Ruchat et al. 2013; Kang et al. 2017). However, translating these findings into clinically relevant biomarkers requires validation, which is currently lacking both globally and in Sub-Saharan Africa. In this study, we evaluated the reproducibility of previously identified genome-wide DNA methylation signatures in South African women with GDM, using bisulfite pyrosequencing. Technical validation confirmed decreased methylation levels at cg23643951 in *CAMTA1* and cg21910650 in *KLHDC3/MEA1*, and increased methylation levels at cg16306629 near *COLEC10*, while these findings were not consistently replicated in the independent biological cohort. Further investigation of differentially methylated CpGs in the technical validation group showed that methylation levels at *KLHDC3/MEA1* were inversely associated with fasting plasma glucose and 1 h OGTT levels, while *COLEC10* was positively correlated with fasting plasma glucose levels, suggesting a potential association with glucose regulation during pregnancy in these women.

In the technical validation cohort, hypomethylation of *CAMTA1* and *KLHDC3/MEA1*, and hypermethylation of *COLECT10* were confirmed in women with GDM compared to women without GDM, using bisulfite pyrosequencing, which was consistent with the original genome-wide findings (Dias et al. 2019). Altered DNA methylation of *CAMTA1* have been widely reported in neurology, oncology and stroke (Henrich et al. 2011; Liu et al. 2022), while no studies have reported differential methylation during GDM, warranting further investigation. *CAMTA1* is a calcium/calmodulin-dependent transcription factor that regulates gene expression through its CG-1 DNA-binding domain (Bouché et al. 2002; Finkler et al. 2007), and has been implicated in cell-cycle regulation and tumour suppression (Henrich et al. 2011). More recently, hypomethylation of *CAMTA1* has been proposed to modulate cytokine expression and calcium-dependent signalling pathways associated with immune and inflammatory regulation (Minskaia et al. 2018). In contrast, one study reported increased methylation of *CAMTA1* in maternal peripheral blood in ectopic pregnancy (Cai et al. 2021), suggesting that *CAMTA1* methylation patterns may differ across distinct pregnancy-related pathological conditions, reflecting disease-specific epigenetic responses rather than a common pregnancy-associated signature. The CpG site annotated to *KLHDC3/MEA1*, lies within a shared GC-rich region linking the two genes in a head-to-head orientation, thus, methylation at this shared region could influence transcription of both genes simultaneously. *KLHDC3* functions in ubiquitin-mediated protein degradation (Zhang et al. 2022), while *MEA1* is primarily testis-specific (Lau et al. 1989), with neither previously linked to metabolic disease. The third CpG site is located in an intergenic region, with the closest regulatory region found within *COLEC10*, which encodes a complement-lectin family protein (Ohtani et al. 1999) with no established role in glucose metabolism. Thus, methylation at these CpG sites warrants further investigation into their potential role in glucose metabolism during pregnancy.

Differential methylation of these CpG sites were not consistently replicated in the larger independent biological cohort. The lack of reproducibility may be attributed to a combination of biological, technical, and population-level factors, each of which has important implications for epigenetic biomarker discovery (Campagna et al. 2021). A primary consideration is that the genome-wide DNA methylation differences between women with and without GDM at individual CpG sites were likely modest (Dias et al. 2019), causing genuine methylation differences to fall below the threshold of statistical detection in a more diverse validation sample. This aligns with the relaxed selection criteria applied during the original genome-wide discovery phase, where candidate CpG sites were identified using an unadjusted *p*-value threshold of < 0.01 in the absence of false discovery rate (FDR) correction (Dias et al. 2019). Furthermore, the discordance in sample selection between the discovery and biological validation cohorts likely further limits reproducibility. The technical validation group was tightly matched for age, BMI, and gestational age, and comprised the same samples used in the original BeadChip array discovery study, thereby minimising confounders known to influence methylation profiles (Burris et al. 2015; Agha et al. 2016), and replicating the original methylation patterns. In contrast, the biological validation group consisted of a broader, more heterogeneous sample group with greater interindividual variability, particularly gestational age. This mismatch between the discovery and replication cohort is a common methodological challenge in epigenetic biomarker research and likely explains why methylation signals detectable under tightly controlled conditions often fail to reach significance when assessed in more representative, heterogeneous settings. Other factors such as platform-related technical differences between the Illumina EPIC BeadChip array and bisulfite pyrosequencing may have contributed to the lack of replication observed. Array-based platforms interrogate methylation at CpG sites across the entire genome, which are subjected to probe-level biases, background noise correction, and normalisation assumptions that differ fundamentally from quantitative pyrosequencing measures at individual CpG loci (Bibikova et al. 2011). While pyrosequencing and BeadChip array measurements generally show good congruence, discordance can arise at individual locus, particularly where methylation differences are small (Noble et al. 2021). Accordingly, differences between platforms have been previously documented in validation studies, where initial findings were not replicated (Binder et al. 2015; Deng et al. 2018), underscoring the importance of careful primer design and amplicon selection, as well as standardised normalisation approaches when comparing methylation measurements across platforms.

Interestingly, differential methylation at *cg21910650* (*KLHDC3/MEA1*) and *cg16306629* (*COLEC10*) was significantly associated with maternal glycaemic parameters within the technical validation group. Methylation at *cg21910650* was inversely correlated with fasting plasma glucose and 1 h OGTT levels, while methylation at *cg16306629* was positively correlated with fasting plasma glucose. This is in line with a previous study conducted by Bouchard et al. (2012), showing an association between lower placental gene-specific methylation and higher maternal glucose concentrations in women with GDM compared to women with normoglycemia (Bouchard et al. 2012). These findings suggest that differential methylation at a particular locus may be associated with glucose tolerance during pregnancy. Future longitudinal follow-up studies of mothers with GDM and their offspring are warranted, to determine whether altered *cg21910650* and *cg16306629* methylation is associated with glucose dysregulation and long-term metabolic health of both mother and child.

To our knowledge, this is the first validation study investigating CpG-specific DNA methylation changes within *CAMTA1*, *KLHDC3/MEA1*, and *COLECT10* in women with and without GDM in a South African population. A notable strength of this study is its population relevant approach, which addresses the under-representation of African populations in GDM-related epigenetic research. Given the increasing burden of GDM in sub-Saharan Africa (Sabbagh et al. 2026), the findings are particularly relevant to the regional public health context. The inclusion of two independent cohorts, together with the confirmation of our previous genome-wide methylation findings in the technical validation group adds further strength to our study (Dias et al. 2019). Additionally, the sample size in our biological validation cohort was larger than those used in previous studies (Binder et al. 2015; Wu et al. 2018), suggesting that our study was sufficiently powered, and that the observed lack of replication could reflect population variability. Although participants were recruited from similar socioeconomic and geographic backgrounds, lifestyle factors, including diet, physical activity, smoking, and alcohol consumption, were not measured. As these factors have been shown to influence DNA methylation (Ryan et al. 2020), residual confounding cannot be excluded. While this study provides important insights for biomarker discovery, several limitations should be considered when interpreting these findings. DNA methylation was quantified in peripheral blood, which consists of a heterogeneous mixture of cell types (Reinius et al. 2012; Fu et al. 2025). Differences in cell-type composition between participants may therefore have influenced methylation patterns at these specific loci. Although cell-type composition was adjusted for statistically in the original genome-wide BeadChip array study, with no significant differences reported (Dias et al. 2019), this adjustment was not performed in the biological validation group, representing an important methodological consideration for future studies. Moreover, maternal DNA methylation in peripheral blood is known to change across the course of gestation (Das et al. 2023). Given the wide range of gestational age in the biological validation group (10 to 27 weeks of gestation), this variability may have introduced additional methylation differences unrelated to GDM status. However, a correlation analysis between gestational age at collection and methylation at the three candidate CpG sites in this sample showed no significant association (data not shown), suggesting that gestational age is unlikely to explain the absence of methylation differences between women with or without GDM in the biological validation group. Future epigenetic studies should adopt standardised protocols for DNA methylation analysis, including sample collection timing, processing, and normalisation procedures, to reduce variability between studies and enable more direct comparisons across populations and methodologies.

## Conclusion

This study provides confirmation of differential methylation at three candidate CpG sites, *cg23643951* (*CAMTA1)*, *cg21910650* (*KLHDC3/MEA1)*, and *cg16306629* (*COLEC10)* in the technical validation group, consistent with our previous genome-wide discovery findings. However, these methylation differences were not reproduced in the larger independent biological cohort of South African women with GDM, highlighting the need for validation in independent cohorts. The association between methylation at *KLHDC3/MEA1* and *COLEC10* with glucose concentrations suggests a potential association with glucose regulation during pregnancy and provides a foundation for future research to validate the clinical utility of *cg23643951*, *cg21910650*, and *cg16306629* DNA methylation as biomarkers for GDM.

## Supplementary Information

Below is the link to the electronic supplementary material.

Supplementary Material 1

Supplementary Material 2

## Data Availability

Included in the paper or Supplementary Information (for raw data, not summary data such as means and variances).All data supporting the findings of this study are available within the paper and its Supplementary Information. Primer sequences are provided as Supplementary material.
