# Supplementary material for: Candidate DNA methylation markers for gestational diabetes: findings from technical and biological validation in a South African population
Source: Mol Genet Genomics. 2026 Oct 26;301(1):205. doi: 10.1007/s00438-026-02528-7 (PMC13710561; doi:10.1007/s00438-026-02528-7)
Supplement: Supplementary file 1 [file 438_2026_2528_MOESM1_ESM.docx]

| Probe ID | Gene symbol | Location | Product length (bp) | Primer sequence | Sequence to analyse |
| --- | --- | --- | --- | --- | --- |
| cg16306629 | *COLECT10* | Chromosome 8 | 119 | Fwd: AGGGAGAGAGATATAGGTAGGA  Rev: CCACTAAAAAATAAAACCCTTTCAATTAC  Seq: ATATAGGTAGGAAATGGG | GTGAGAGGAT GYGGTGAGGG GAGGTGAGGG AYGGTTAAGA AAAATTTTGT AGGATTT |
| cg21910650 | *KLHDC3/MEA1* | Chromosome 6, intron 1 | 122 | Fwd: AAGGGAGGATGGAAGATTTTTA  Rev: AACCCCAACAAAAAACTACTCTCA  Seq: AGGATGGAAGATTTTTATTT | AGGATGGAAG ATTTTTATTT TTAAGTTTAT YGATTTTTTA ATAGTTTTTA TAAGTTTAGA TTTAA |
| cg23643951 | *CAMTA1* | Chromosome 1, intron 4 | 256 | Fwd: GTAGGTTTGTTGGTTGTTTTTAGAA  Rev: CATATAAACATCCTCTACTTAACACATAC  Seq: AGTGTTTAAATTGTTTGTATAAAT | TATGTYGTTT ATGTYGAGTA TTGGTTTTTT TT |

Table S1. Primer design for CpG-specific DNA methylation using pyrosequencing
