# Supplementary material for: Candidate DNA methylation markers for gestational diabetes: findings from technical and biological validation in a South African population
Source: Mol Genet Genomics. 2026 Oct 26;301(1):205. doi: 10.1007/s00438-026-02528-7 (PMC13710561; doi:10.1007/s00438-026-02528-7)
Supplement: Supplementary file 2 [file 438_2026_2528_MOESM2_ESM.docx]

A.

B.

C.

**Figure S1. Quality control for validation assays using pyrosequencing**. Bisulfite conversion efficiency and assay performance were evaluated using commercially available methylation standards (0%, 10%, 25%, 50%, 75% and 100% methylation) and bisulfite conversion controls. Validation was performed for pyrosequencing assays targeting A) CAMTA1, B) MEA1/KLHDC3 and C) COLECT10. Assays demonstrated high linearity and reproducibility across the full methylation range, confirming assay reliability.
